# Decision‐making accuracy of soccer referees in relation to markers of internal and external load

**DOI:** 10.1002/ejsc.12096

**Published:** 2024-03-18

**Authors:** Gary P. McEwan, Viswanath B. Unnithan, Chris Easton, Andrew J. Glover, Rosie Arthur

**Affiliations:** ^1^ Division of Sport and Exercise University of the West of Scotland Glasgow UK; ^2^ Division of Psychology Sociology and Education Queen Margaret University Edinburgh UK

**Keywords:** assessment, cognition, performance, physiology, team sport

## Abstract

This study examined the relationships between the decision‐making performances of soccer referees and markers of physiological load. Following baseline measurements and habituation procedures, 13 national‐level male referees completed a novel Soccer Referee Simulation whilst simultaneously adjudicating on a series of video‐based decision‐making clips. The correctness of each decision was assessed in relation to the mean heart rate (HR), respiratory rate (RR), minute ventilation (VE), perceptions of breathlessness (RPE‐B) and local muscular (RPE‐M) exertion and running speeds recorded in the 10‐s and 60‐s preceding decisions. There was a significant association between decision‐making accuracy and the mean HR (*p* = 0.042; V_C_ = 0.272) and RR (*p* = 0.024, V_C_ = 0.239) in the 10‐s preceding decisions, with significantly more errors observed when HR ≥ 90% of HR_max_ (OR, 5.39) and RR ≥ 80% of RR_peak_ (OR, 3.34). Decision‐making accuracy was also significantly associated with the mean running speeds performed in the 10‐s (*p* = 0.003; V_C_ = 0.320) and 60‐s (*p* = 0.016; V_C_ = 0.253) preceding decisions, with workloads of ≥250 m·min^−1^ associated with an increased occurrence of decisional errors (OR, 3.84). Finally, there was a significant association between decision‐making accuracy and RPE‐B (*p* = 0.021; V_C_ = 0.287), with a disproportionate number of errors occurring when RPE‐B was rated as *“very strong”* to *“maximal”* (OR, 7.19). Collectively, the current data offer novel insights into the detrimental effects that high workloads may have upon the decision‐making performances of soccer referees. Such information may be useful in designing combined physical and decision‐making training programmes that prepare soccer referees for the periods of match play that prove most problematic to their decision‐making.

## Highlights

- Soccer referees exhibited an increased likelihood of making a decisional error when: (1) HR ≥ 90% of HR_max_; (2) RR ≥ 80% of RR_peak_; (3) RPE‐B was rated as “*very strong”* to “*maximal”* (75–100 au); and (4) running speeds ≥250 m·min^−1^ were performed in the 10‐s prior to a decision.
- In contrast, the accuracy of the referees' decisions was not related to mean measures of HR, RR, or V̇E obtained in the 60‐s preceding decisions.
- The relationship between the physiological and decision‐making aspects of refereeing performance therefore appears to be a transient one, whereby high internal and external loads experienced immediately prior to infringements may compromise the correctness of the decision.
- Such information may be used to guide the design and delivery of training programmes aimed at preparing soccer referees for the periods of match play that prove most problematic to their decision‐making.

## 1 INTRODUCTION

Soccer referees are tasked with ensuring that match play is contested in accordance with the Laws of the Game (IFAB, 2022). A key aspect of this responsibility is to identify incidents of foul play, with ∼26 fouls awarded per match (Mallo et al., 2012). To enhance their perception of potential infringements, referees must remain close to play, adopting suitable viewing positions without obstructing the ball or players (Mallo et al., 2012). Elite referees therefore cover total distances (TD) of 9–12 km during competitive matches, with high‐speed running (HSR) ≥18 km·h^−1^ accounting for ∼1800 m (Krustrup et al., 2009; Mallo et al., 2009). The internal loads elicited during match play are also considerable with elite referees attaining mean match heart rates (HR) of ∼85% of their maximal (HR_max_; Krustrup et al., 2009; Mallo et al., 2009). A key challenge facing referees is therefore the necessity to undertake complex perceptual‐cognitive processes in combination with elevated levels of physiological stress. In considering the implications that high physiological loads may have on the decision‐making process, the relationship between these facets of performance requires investigation (Weston et al., 2012).

In the broad domain of sport and exercise science, many studies have explored the impact of acute exercise upon perceptual‐cognitive performance (Basso & Suzuki, 2017; McMorris & Hale, 2012). Synthesising the available evidence, narrative and meta‐analytic reviews have generally concluded that moderate‐intensity exercise can increase processing speeds, with little effect on response accuracy (Basso & Suzuki, 2017; McMorris & Hale, 2012). The findings of individual studies do however display notable variability, with reports of both positive and negative effects. Such variability is likely due to differing moderating variables such as the type, intensity, and complexity of the exercise stimulus and perceptual‐cognitive task (McMorris & Hale, 2012). For instance, enhancements in a simple multiple‐choice reaction time test were previously noted amongst elite soccer players during a simulated match (Wiśnik et al., 2011). Recent data does however indicate that performance during more complex, soccer‐specific tasks may decline with increasing physical loads (Alder et al., 2021). Considering the intricate nature of soccer refereeing and the need to make complex judgements during high‐intensity, intermittent activity, the ability to extend findings obtained from other domains to soccer officials is perhaps limited.

Efforts to explore the impact of elevated levels of physiological load upon the decision‐making of team sport officials have thus far been relatively scarce (Bloß et al., 2020). Recently, Pizzera and colleagues (2022) examined the performances of soccer referees during a video‐based decision‐making task administered at rest and whilst running at 60% and 80% of maximal oxygen uptake (V̇O_2max_). It was reported that physical exertion had no influence upon decision‐making, with decision‐making performance remaining comparable between conditions. Several studies have also explored this association in situ, often ascertaining the officials' decision‐making accuracy across distinct fixed‐time epochs and correlating this against the physiological loads imposed during that same period. In an investigation by Mascarenhas and colleagues (2009), for example, the decision‐making accuracy of seven soccer referees was unrelated to their average running speed and TD across 15‐min match segments. A similar investigation amongst Rugby League referees found no association between decision‐making performance and mean HR or HSR distance when explored across 10‐min intervals (Emmonds et al., 2015). While these data may indicate that decision‐making is unrelated to physical load, the analytical approaches adopted are likely insufficient to assess the intricacies of such a relationship. Indeed, current evidence suggests that discrete fixed‐time epochs may underestimate physical match demands as compared to rolling averages (Fereday et al., 2020). Aggregating data over prolonged epochs may also conceal acute periods of high physiological stress that could impact decision‐making. Although high‐intensity episodes last only seconds (Barbero‐Álvarez et al., 2012), it is plausible that such exertions may temporarily impair an official's decision‐making. Several theoretical frameworks indeed exist to support a possible negative effect of physical stress on decision‐making. One line of thought relates to the attentional control theory, which posits that increasing task demands may place an additional strain on an official's limited attentional resources (Eysenck et al., 2007). That is, at higher levels of physical exertion, referees may be inclined to direct greater attention towards addressing physiological perturbations and ensuring their own postural stability, thereby diverting attention away from the incident at the critical moment. Examining the correctness of decisions immediately following shorter, acute periods of high physical stress may therefore permit a better understanding of the relationship that exists between the physical and decision‐making performances of match officials.

A previous investigation amongst soccer referees found no association between the officials' decision‐making accuracy and the TD covered 30 s before an incident (Riiser et al., 2019). Similar observations were noted amongst Australian Football umpires, with decision‐making accuracy found to be unrelated to the mean running speeds recorded 30 s to 5 min prior to the decision (Elsworthy et al., 2014). Higher relative running speeds performed 5 s before a decision were however associated with a greater occurrence of decisional errors (Elsworthy et al., 2014). While an enhanced physiological demand could explain such findings, this remains speculative as no measures of internal load were obtained. As isolated and repeated bouts of HSR frequently precede crucial match moments like goals and goal scoring opportunities (Martínez‐Hernández et al., 2022), additional research is required to examine their impact on the decision‐making accuracy of soccer referees. Indeed, as decisional errors can greatly impact match outcomes, deepening our understanding of the relationship between these facets of performance represents an important step if researchers and practitioners are to positively influence the decision‐making performances of soccer referees. That is, by understanding how the physical demands of match play influence the judgements made by soccer officials, training interventions that replicate the types of situations and contexts that prove problematic to their decision‐making may be developed. The purpose of the present study was therefore to assess the decision‐making performances of soccer referees with respect to the internal and external loads recorded in the moments immediately preceding decisions. Considering the literature discussed, the following hypotheses are proposed: (1) higher internal and external loads recorded in the 10‐s and 60‐s preceding decisions will be associated with a greater occurrence of decisional errors than lower loads; and (2) stronger associations would be identified using smaller (10‐s) than larger (60‐s) epochs.

## 2 METHODS

### 2.1 Participants

Thirteen soccer referees (age: 30.4 ± 4.1 years; stature: 177.5 ± 7.5 cm; body mass: 76.9 ± 10.2 kg; and V̇O_2max_: 53.5 ± 3.5 mL·kg·min^−1^) from the Scottish Football Association (SFA) participated in the current investigation. Participants possessed 7.9 ± 2.0 years of officiating experience and had officiated at a national level for 4.5 ± 1.9 years. On average, participants trained for 3.7 ± 0.8 h/week and officiated 1–2 matches per week within the Scottish Championship and/or Scottish League One. Informed written consent was obtained from all participants prior to testing, and the study received institutional ethical approval.

### 2.2 Preliminary measurements and habituation

Referees attended the laboratory twice during the in‐season (October to December), with a maximum of 7 days separating trials. Participants abstained from strenuous exercise in the 48‐h preceding each session. Trials were conducted at a similar time of day (±1h) under standardised environmental conditions (temperature: 19°C; relative humidity: 40%).

Participants' V̇O_2max_ and HR_max_ were established during a ramp incremental test on a motorised treadmill (Woodway PPS 55sport‐I, USA). Following a standardised warm‐up, participants commenced running at 8 km·h^−1^ for 2 minutes, with the speed increased by 1 km·h^−1^ every minute until 15 km·h^−1^. Thereafter, speed remained constant with the gradient increased by 1% every minute until volitional exhaustion (Sperlich et al., 2015). Participants were instructed to perform to the best of their ability and received verbal encouragement throughout. Participants' HR and respiratory variables were monitored throughout via HR telemetry (Polar H10, Finland) and breath‐by‐breath gas analysis (Jaeger Oxycon Pro, Germany), respectively. V̇O_2max_ was considered the highest V̇O_2_ value recorded using 15‐breath rolling averages with HR_max_ defined as the highest value recorded. Achievement of at least two of the following criteria confirmed attainment of V̇O_2max_: (1) plateau in V̇O_2_ despite an increase in speed; (2) HR within ±10 beats·min^−1^ of age‐predicted HR_max_ and (3) a respiratory exchange ratio ≥1.10. Additionally, respiratory rate (RR) and minute ventilation (V̇E) were averaged on a 5‐s basis, with the highest value recorded in a 20‐s period retained as RR_peak_ and V̇E_peak_, respectively (Buchheit et al., 2009).

Upon completion, participants received ∼15 min recovery before being habituated to the Soccer Referee Simulation (SRS) and main experimental procedures. During the habituation process, participants were acclimated to the stochastic, intermittent velocity profile of the SRS and completed 5 practice video clips. Crucially, all participants were familiar with video‐based testing having performed this regularly as part of their training within the previous 2 years. Participants had not however been exposed to the specific clips used within the present study during either training or previous research.

### 2.3 Soccer referee simulation

During the main trial, the SRS was performed on a programmable motorised treadmill (Woodway PPS 55sport‐I, USA). The validity and reliability of the SRS have been described previously (McEwan et al., 2023). Briefly, two ∼16‐min blocks interspersed with a 90‐s passive recovery period were performed (see Supplemental Figure for schematic of SRS). The protocol incorporated varying periods of standing, walking (6 km·h^−1^), jogging (11 km·h^−1^), cruising (15 km·h^−1^), and HSR (18 km·h^−1^), with the frequency and duration of activities reflecting previous literature (Barbero‐Alvarez et al., 2012; Krustrup et al., 2009). Considering the impracticalities of changing speed every few seconds on a motorised treadmill, the frequency and duration of occurrences were manipulated by a factor that resulted in an activity change every 6–23 s. This resulted in 145 activity changes, with the rate of acceleration/deceleration between changes set at 2 m·s^−2^. Previous data suggest that ∼36% of the accelerations/decelerations performed by elite referees during matches are performed at rates of 1.5–2.5 m·s^−2^ (Castillo et al., 2018). Activity changes were communicated to participants via a visual countdown displayed on a large monitor positioned in front of the treadmill.

Throughout the SRS, each referee was presented with 10 soccer‐specific decision‐making clips on a 40‐inch monitor (NEC MultiSync LCD4010, Japan) positioned ∼2 m in front of the treadmill. The number of clips presented to each participant reflected the relative frequency of fouls that occur during a match (Mallo et al., 2012). Video clips were sourced from the Refereeing Department of the Scottish Football Association and represented potential foul‐play incidents from club and international European matches. Video clips were selected following consultation with two former international referees (combined to total of 26 years on the FIFA list) and were deemed acceptable if they: (1) depicted a foul‐play scenario in the central area of the field whereby input from the assistant referee would be limited (Mallo et al., 2012); (2) omitted the in‐game official's decision; and (3) were of high visual quality and presented from an in‐game perspective. Regarding this latter criterion, whilst clips were sourced from match broadcast footage, they were presented from a vantage point that closely resembled a referee's perspective on the field, rather than a distant grandstand position. We chose not to include potential penalty decisions as these are one of the four moments in which referees may receive assistance from the Video Assistant Referee (VAR). To control for the impact of contextual factors upon participants' decisions, the elapsed time of the match, the score, and the background sound were removed. Reference decisions were determined by the two former referees who independently assessed each clip as per Law 12 (Fouls and Misconduct) of the Laws of the Game (IFAB, 2022). To facilitate their decision‐making, video clips were able to be viewed multiple times in both real time and slow motion (Spitz et al., 2018). Complete agreement was exhibited between experts for each clip with decisions deemed to be of a similar level of difficulty. The following reference decisions were reached: no foul (*n* = 26), foul without caution (*n* = 39), foul with yellow card (*n* = 39) and foul with red card (*n* = 26). Of the 10 video clips administered during each SRS, 5 were administered during a stand phase and 5 were administered during a jog phase, with each of the five different locomotor activities immediately preceding a clip on two occasions. Upon viewing, referees made one of the following four decisions: no foul, foul, foul with yellow card, or foul with red card (Spitz et al., 2018). Participants' decisions were assessed against the reference decisions and were categorised as correct or incorrect.

Throughout each trial, participants' HR and respiratory variables were measured as previously described. As fixed epochs can underestimate physiological demands (Fereday et al., 2020), HR data were processed using 10‐s and 60‐s rolling averages, with the mean HR recorded prior to each decision retained for analysis. Mean HR data were expressed in relative terms as a percentage of participants' HR_max_ and were classified into four distinct zones: 60%–69% HR_max_, 70%–79% HR_max_, 80%–89% HR_max,_ and >90% HR_max_ (Edwards, 1993). The mean RR and V̇E recorded in the 10‐s and 60‐s preceding each decision was also calculated and retained as a percentage of participants' RR_peak_ and V̇E_peak_, respectively (Buchheit et al., 2009).

Using the CR100 scale, participants provided differential ratings of perceived exertion (RPE) to delineate between perceptions of breathlessness (RPE‐B) and local muscular (RPE‐M) exertion (Weston et al., 2015). To control for the potential influence of acute fatigue upon RPE, ratings were collected during the jog phase preceding each decision and were obtained in a counterbalanced manner to eliminate order effects (Weston et al., 2015). Participants were habituated with this scale during the preliminary session and received instruction on how to appraise differential RPE. Specifically, participants were informed that RPE‐B depends mainly on the breathing rate and/or heart effort, and RPE‐M depends mainly on the strain and exertion in the lower limbs (McLaren et al., 2020). Differential RPE were subsequently classified into four arbitrary categories: *“nothing at all* to *moderate”* (0–25 au), *“moderate* to *strong”* (26–50 au), *“strong* to *very strong”* (51–75 au), and *“very strong* to *maximal”* (76–100) (Lovell et al., 2020).

The average running speeds performed in the 10‐s and 60‐s preceding decisions were also calculated. To aid the practical application of the findings, running speeds were expressed as a relative measure (m·min^−1^) and were categorised as: <150 m·min^−1^, 150–199 m·min^−1^, 200–249 m·min^−1^, and ≥250 m·min^−1^.

### 2.4 Data analysis

To explore associations between decision‐making accuracy and physiological load, the number of correct and incorrect decisions was calculated for each of the intensity bandwidths of the physiological variables. For measures of HR, for example, we established the number of correct and incorrect decisions made within each HR zone (i.e., 60%–69%, 70%–79%, 80%–89%, and >90% HR_max_). Chi‐squared tests of independence were subsequently performed to examine if decision‐making accuracy was uniformly distributed across the pre‐defined intensity categories (Nevill et al., 2002). The magnitudes of associations were assessed using Cramer's V (V_C_) effect sizes and interpreted as: trivial (<0.1), small (0.1–0.29), moderate (0.3–0.49), or large (≥0.5) (Cohen, 1992). To identify cells where observed counts deviated from an expected equal distribution, standardised residuals (SR) were calculated with values of less than −2 and greater than 2 considered statistically significant (Agresti, 2007). Odds ratios (OR), with uncertainty expressed as 95% CI, were subsequently calculated to examine the likelihood of making a decisional error associated with each categorical variable. Statistical procedures were completed using SPSS 26.0 (IBM, USA), with statistical significance set at *p* < 0.05.

## 3 RESULTS

### 3.1 Decision‐making accuracy

Of the 130 decisions assessed, 31 errors were made, representing a mean decision‐making accuracy of 76.2 ± 11.2% (range: 60% – 90%).

## 4 PHYSIOLOGICAL AND PERCEPTUAL RESPONSES

The physiological and perceptual responses elicited during the 10‐s and 60‐s preceding decisions are summarised in Table 1.

**TABLE 1:** Physiological and perceptual responses elicited during the 10‐ and 60‐s preceding decisions.

	10‐s	60‐s
Heart Rate (HR)		
Mean HR (% of HR_max_)	81.7 ± 6.3	79.4 ± 6.9
Respiratory variables		
V̇O_2_ (% of V̇O_2max_)	64.5 ± 11.1	65.4 ± 9.9
RR (% of RR_peak_)	68.2 ± 13.8	67.0 ± 11.1
V̇E (% of V̇E_peak_)	51.0 ± 8.5	49.6 ± 7.0
Differential RPE		
RPE‐B (au)	/	36 ± 22
RPE‐M (au)	/	28 ± 19

*Note*: Data presented as mean ± SD.

Abbreviations: HR_max_, maximal heart rate; RPE‐B, breathlessness; RPE‐M, muscular; RRpeak, peak respiratory rate; V̇E_peak_, peak minute ventilation; V̇O_2max_, maximal oxygen uptake capacity.

### 4.1 Decision‐making accuracy in relation to heart rate

**FIGURE 1:**
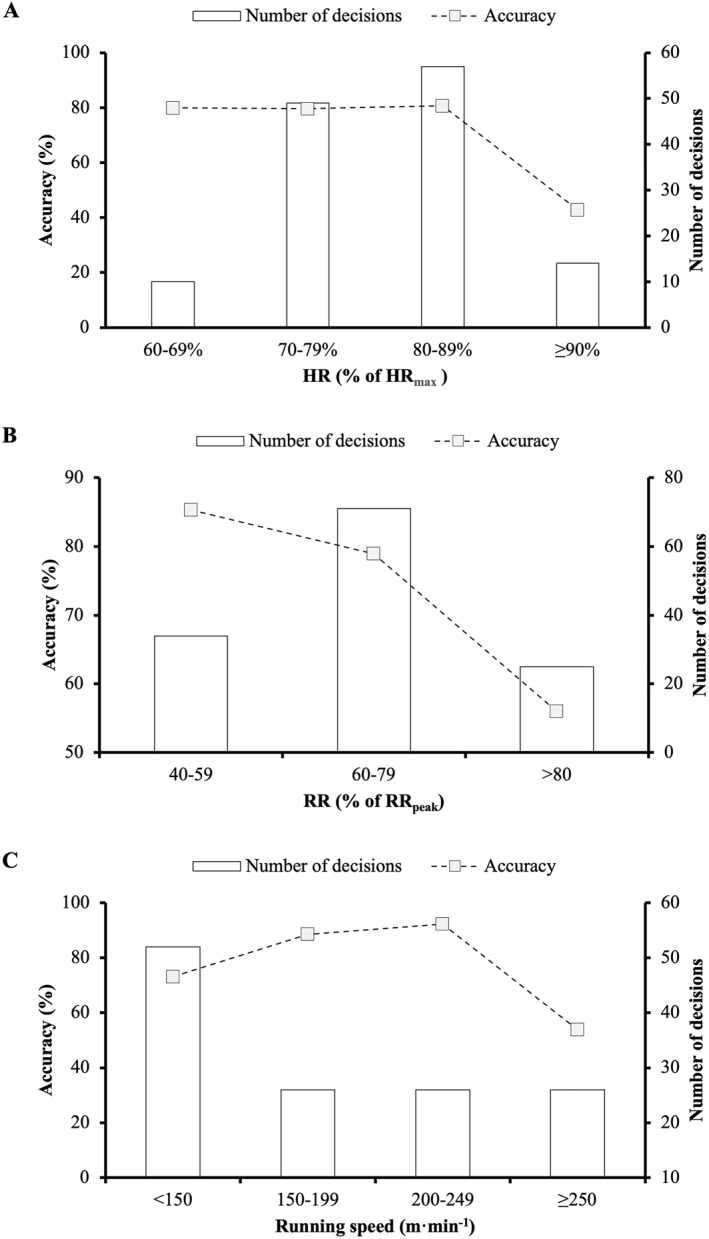
Decision‐making accuracy in relation to mean heart rate (HR; A), respiratory rate (RR; B) and running speed (C) in the 10 s preceding each decision.

**FIGURE 2:**
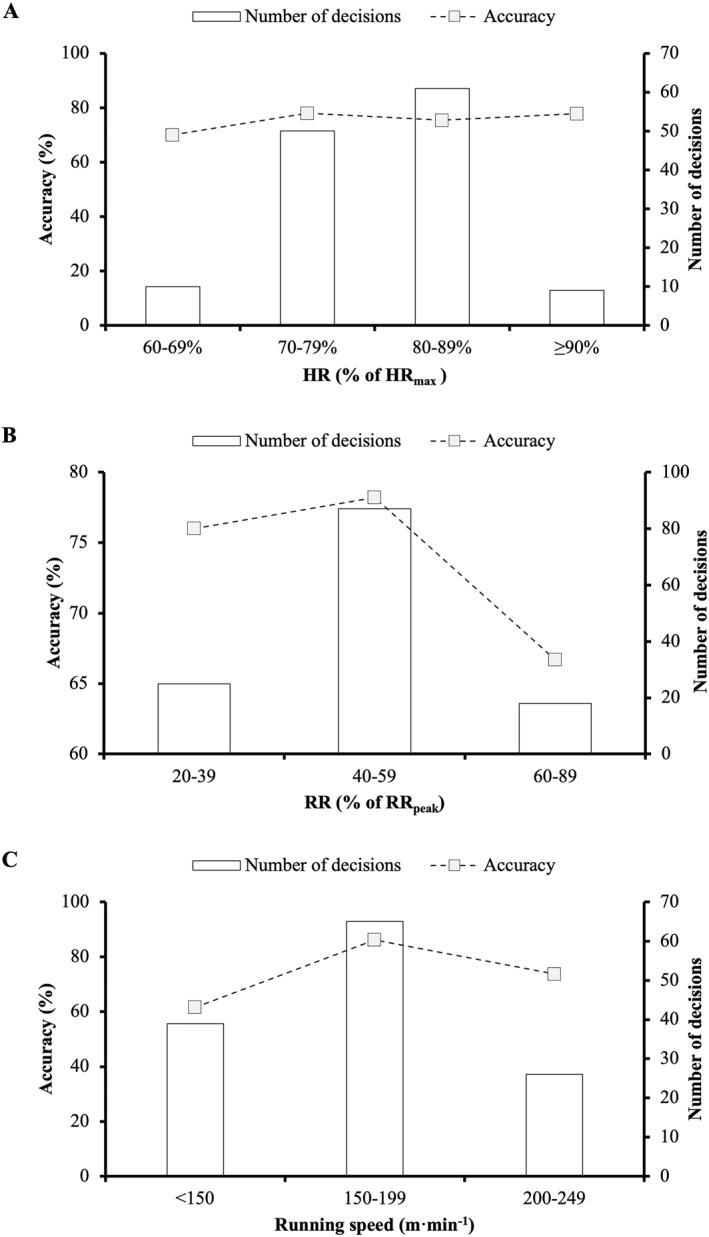
Decision‐making accuracy in relation to mean heart rate (HR; A), respiratory rate (RR; B) and running speed (C) in the 60 s preceding each decision.

There was a significant association between decision‐making accuracy and mean HR in the 10‐s preceding a decision (*χ* ^2^_(3)_ = 8.181, *p* = 0.042, and V_C_ = 0.272), with significantly more errors observed when HR ≥ 90% of HR_max_ (SR, 2.6; OR, 5.39, and 95% CI, 1.70–17.07) (Figure 1A). No association was observed between decision‐making accuracy and mean HR in the 60‐s preceding a decision (*χ* ^2^_(3)_ = 0.325, *p* = 0.955, and V_C_ = 0.051) (Figure 2A).

### 4.2 Decision‐making accuracy in relation to respiratory variables

There was a significant association between decision‐making accuracy and mean RR in the 10‐s preceding a decision (*χ* ^2^_(2)_ = 7.445, *p* = 0.024, and V_C_ = 0.239), with significantly more errors observed when RR ≥ 80% of RR_peak_ (SR, 2.1; OR, 3.34, and 95% CI, 1.32–8.45) (Figure 1B). No association was observed between decision‐making accuracy and mean RR in the 60‐s preceding a decision (*χ* ^2^_(2)_ = 1.085, *p* = 0.581, and V_C_ = 0.091) (Figure 2B).

No association was observed between decision‐making accuracy and mean V̇E in the 10‐s (*χ* ^2^_(2)_ = 4.144, *p* = 0.126, and V_C_ = 0.179) or 60‐s (χ^2^_(2)_ = 0.955, *p* = 0.620, and V_C_ = 0.086) preceding a decision.

### 4.3 Decision‐making accuracy in relation to differential RPE

**FIGURE 3:**
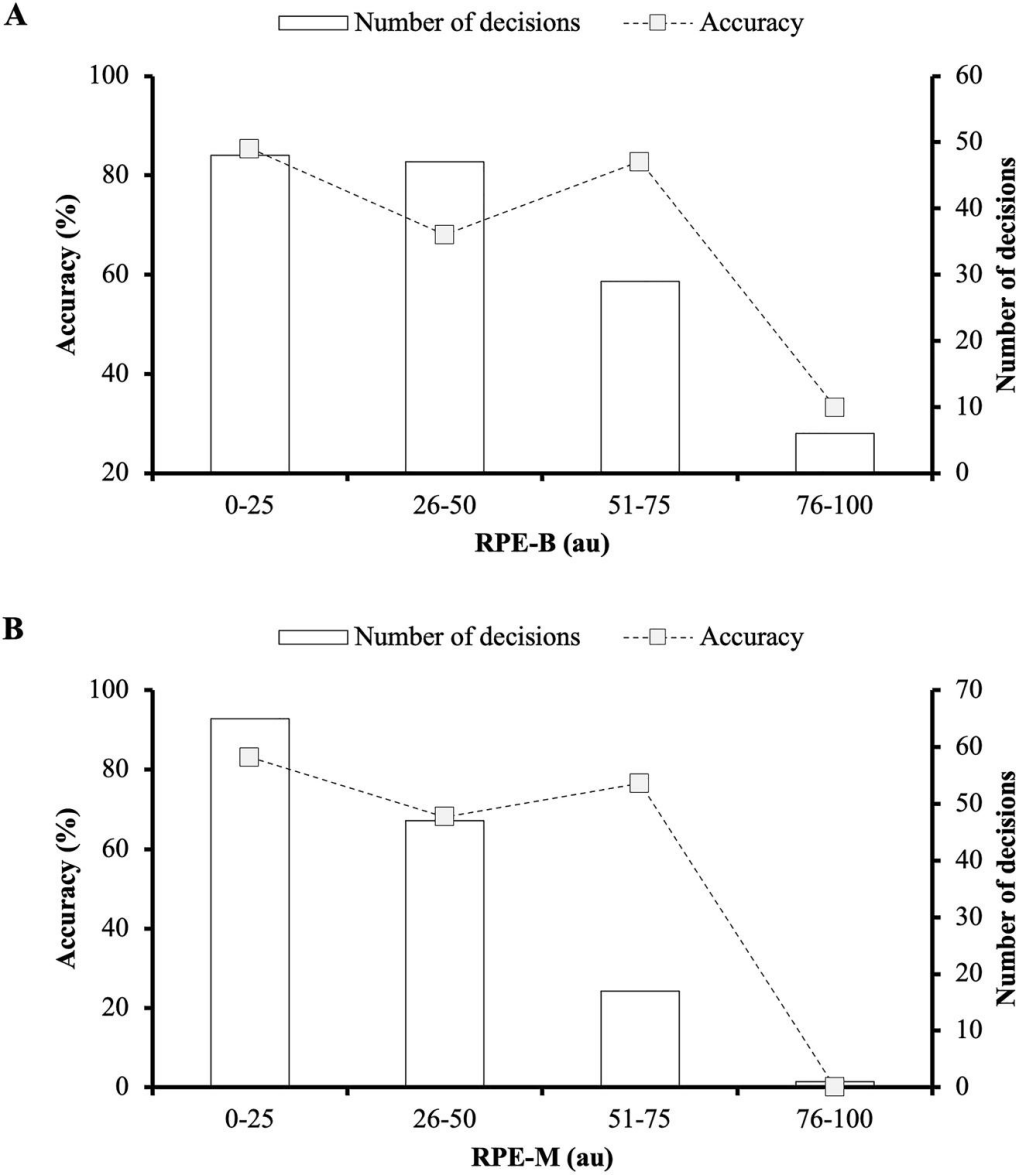
Decision‐making accuracy in relation to perceptions of breathlessness (RPE‐B; A) and local muscular (RPE‐M; B) exertion.

There was a significant association between decision‐making accuracy and RPE‐B (*χ* ^2^_(3)_ = 9.773, *p* = 0.021, and V_C_ = 0.287), with significantly more errors observed when RPE‐B was rated “*very strong*” to “*maximal*” (Figure 3A). No association was observed between decision‐making accuracy and RPE‐M (*χ* ^2^_(3)_ = 6.296, *p* = 0.098, and V_C_ = 0.225) (Figure 3B).

### 4.4 Decision‐making accuracy in relation to the running speed prior to decision

There was a significant association between decision‐making accuracy and mean running speed in the 10‐s preceding a decision (*χ* ^2^_(3)_ = 13.651, *p* = 0.003, and V_C_ = 0.320), with significantly more errors observed when running speeds were ≥250 m·min^−1^ (SR, 2.3; OR, 3.84, and 95% CI, 1.53–9.60) (Figure 1C). Decision‐making accuracy was also significantly associated with mean running speed in the 60‐s preceding a decision (*χ* ^2^_(3)_ = 8.301, *p* = 0.016, and V_C_ = 0.253) (Figure 2C).

## 5 DISCUSSION

This study explored the association between various markers of internal and external load and the accuracy of foul‐play decisions of soccer referees. Collectively, the current data offer novel insights into the detrimental effects that high workloads may have upon soccer referees' decision‐making. Specifically, officials were more likely to make an incorrect decision when: (1) HR ≥ 90% of HR_max_; (2) RR ≥ 80% of RR_peak_; (3) RPE‐B was rated as *very strong* to *maximal* (75–100 au); and (4) running speeds ≥250 m·min^−1^ were performed. Such information may be used to inform the design and delivery of training programmes aimed at preparing soccer referees for the periods of match play that prove most problematic to their decision‐making.

Our data indicate that the accuracy of officials' decisions was not related to the mean HR observed 60 s prior to the decision. Yet, when examined in relation to the HR exhibited 10 s prior, referees were 5.4 times more likely to make an error when HR ≥ 90% of HR_max_. This finding stands in contrast to a recent investigation showing that the decision‐making performances of sub‐elite soccer referees remained unaltered by increasing levels of physical exertion (Pizzera et al., 2022). An earlier study by Emmonds et al. (2015) also found no association between the decision‐making accuracy of Rugby League officials and metrics of mean and peak HR. While speculative, such discrepancies might stem from differences in the exercise stimulus and the analytical approaches adopted. Firstly, the protocol employed by Pizzera et al. (2022) sought to replicate mean match play intensities, with referees running at steady intensities of 60% and 80% of V̇O_2max_. Given this approach, it is unlikely that these officials reached the upper intensities observed in the current study to influence decision‐making (i.e., ≥90% of HR_max_). Secondly, both studies assessed the accuracy of the official's decisions across distinct fixed‐time epochs, contextualising these against the physiological loads recorded during that same period. A more nuanced analysis was however performed in the current investigation, assessing the correctness of each decision in relation to the internal loads exhibited immediately preceding the decision. While further research is warranted to identify the underlying mechanisms of this relationship, the disparities noted between the 10‐s and 60‐s epochs may suggest that the relationship between the physiological and decision‐making aspects of refereeing performance is a transient one, whereby high internal loads at the time of an infringement may compromise decision accuracy. Regarding the dual‐task paradigm, it has been posited that cognitive performance is likely impaired during high‐intensity exercise when both cognitive and physiological demands peak and converge simultaneously (Sudo et al., 2022). As referees attain HRs ≥90% of HR_max_ during competitive matches (Mallo et al., 2009), such findings hold important implications for soccer referees' decision‐making, particularly when the most intense periods of match play align with the requirement to make a decision.

Another novel aspect of the present study concerns the assessment of decision‐making in relation to perceived levels of central and peripheral exertion. When RPE‐B was rated as *very strong* to *maximal* (75–100 au), officials were 7.2 times more likely to make a decisional error. As RPE‐B is driven by sensory and affective cues like RR and HR (Borg et al., 2010), these findings are perhaps unsurprising given the reduced decision‐making accuracy observed when RR and HR were ≥80% and ≥90% of RR_peak_ and HR_max_, respectively. Moreover, earlier studies have shown that sensations of breathlessness can induce heightened anxiety levels amongst highly trained athletes, particularly at greater levels of ventilation (Faull et al., 2016). Thus, it appears plausible that under high levels of cardiorespiratory stress, attention might have shifted away from the decision at the critical moment, with officials focusing more on stabilising their breathing rate (McEwan et al., 2018). Alternatively, it has been suggested that the onset of hypocapnia, induced by hyperventilation during high‐intensity exercise, may lead to a reduction in cerebral blood flow, thus impacting cognitive performance (Smith & Ainslie, 2017). Nevertheless, this remains speculative, and additional research is required to identify the mechanisms through which high internal loads may compromise the decision‐making performances of soccer officials.

In comparison, the correctness of the officials' decisions was unrelated to measures of RPE‐M. It should however be acknowledged that only one decision was made when RPE‐M was rated as *very strong* to *maximal*. While soccer refereeing is typically associated with a greater perceived peripheral demand (Castillo et al., 2018), the current cohort reported higher levels of perceived respiratory exertion. Since RPE‐M depends mainly on the strain and exertion in the lower limbs, these findings may partly reflect the exclusion of referee‐specific movements and changes of direction (Hader et al., 2014). Moreover, moderate correlations have previously been reported between RPE‐M and GPS‐derived measures of external load such as HSR (Weston et al., 2015). The lower RPE‐M observed in the current study may therefore simply be a result of the shorter duration of the SRS protocol compared to match play. Whilst HR measures remain relatively stable across 15‐min periods of match play (Krustrup et al., 2009), levels of neuromuscular fatigue develop progressively throughout a match (Goodall et al., 2017). Although it was beyond the scope of the present study, future research may wish to explore the effect that match‐related fatigue has on the decision‐making performances of soccer officials, particularly towards the latter stages of match play.

Another key finding concerns the significant associations observed between decision‐making accuracy and the mean running speeds performed 10 and 60 s prior to a decision. Specifically, officials were 3.8 times more likely to make an error having performed external workloads of ≥250 m·min^−1^ in the 10‐s preceding the decision. These findings align with those of Elsworthy and colleagues (2014) who noted higher running speeds performed by Australian Football umpires in the 5 s before a decision to have increased the likelihood of an error being made. Conversely, decision accuracy amongst 11 Norwegian soccer referees appeared unrelated to the TD covered 10 and 30 s before a decision (Riiser et al., 2019). Nonetheless, only 6 of the foul‐play decisions made by these officials were deemed incorrect, representing an error rate of only 1.7%. While the reasons for this low error rate remain unclear, the failure of Riiser et al. (2019) to observe any associations between the physical and decision‐making performances of their cohort perhaps indicates a lack of statistical power.

The current study is not without some limitations. Most important is the performance of a simulated match protocol and therefore a reduced level of ecological validity. Another limitation relates to the relatively small sample of preselected video clips, although the frequency of clips was consistent with the relative number of fouls that occur during match play (Mallo et al., 2012). Additionally, video‐based testing has been shown to possess high levels of construct and discriminant validity amongst team sport officials (Spitz et al., 2018). While enhanced ecological validity could be achieved within naturalistic settings, the current approach allowed for the physical and decision‐making performances of soccer officials to be assessed in isolation from contextual match factors that may further exacerbate decision‐making accuracy. Lastly, although officials in the current study were of a national level, future research is required to confirm whether our findings are reproducible within elite populations.

## 6 CONCLUSIONS AND PRACTICAL IMPLICATIONS

The present findings suggested that the decision‐making performances of soccer referees may be compromised under high cardiorespiratory stress, as evidenced by a marked increase in decisional errors when HR was ≥90% of HR_max_ and RR was ≥80% of RR_peak_ in the 10‐s preceding the decision. Errors were also more frequent when RPE‐B was rated as *very strong* to *maximal* (75–100 au) and when high running speeds (≥250 m·min^−1^) were sustained in the 10 s prior to the decision. These observations therefore reaffirm the importance of high levels of aerobic fitness amongst soccer referees (Castagna et al., 2019). Whilst well‐developed physical capacities enable soccer referees to keep up with play and adopt suitable viewing positions, our findings suggest that high levels of physical fitness may also be crucial in managing or mitigating the sudden spikes in physiological stress that can hinder decision‐making. It therefore stands to reason that soccer officials should continue to apportion ample time to their physical conditioning. However, in considering the direct impact that acute periods of high physiological stress had on the correctness of the referees' decisions, it would also appear important that officials prepare for such challenges during training (Kittel et al., 2019, 2023). Yet, as highlighted recently (McEwan et al., 2022), the physical and decision‐making abilities of soccer referees are typically developed in isolation. Considering this, practitioners may wish to use the present data to inform the design of combined physical and decision‐making training sessions that help to prepare soccer officials for the periods of match play that prove most problematic to their decision‐making.

## CONFLICT OF INTEREST STATEMENT

The authors report no conflict of interest.

## Supporting information

Figure S1

## References

[ejsc12096-bib-0001] Agresti, A. 2007. An Introduction to Categorical Data Analysis, 21–64. Hoboken: Wiley.

[ejsc12096-bib-0002] Alder, David , David P. Broadbent , and Jamie Poolton . 2021. “The Combination of Physical and Mental Load Exacerbates the Negative Effect of Each on the Capability of Skilled Soccer Players to Anticipate Action.” Journal of Sports Science 39(9): 1030–1038. 10.1080/02640414.2020.1855747. 33274696

[ejsc12096-bib-0003] Barbero‐Álvarez, JoséC. , Daniel A. Boullosa , Fábio Y. Nakamura , Germán Andrín , and Carlo Castagna . 2012. “Physical and Physiological Demands of Field and Assistant Soccer Referees during America’s Cup.” The Journal of Strength & Conditioning Research 26(5): 1383–1388. 10.1519/JSC.0b013e31825183c5. 22395268

[ejsc12096-bib-0004] Basso, Julia C. , and Wendy A. Suzuki . 2017. “The Effects of Acute Exercise on Mood, Cognition, Neurophysiology, and Neurochemical Pathways: A Review.” Brain Plasticity 2(2): 127–152. 10.3233/NPL-160040. 29765853 PMC5928534

[ejsc12096-bib-0005] Bloß, N. , J. Schorer , F. Loffing , and Büsch . 2020. “Physical Load and Referees’ Decision‐Making in Sports Games: A Scoping Review.” Journal of Sports Science and Medicine 19(1): 149–157. 32132838 PMC7039031

[ejsc12096-bib-0006] Borg, E. , G. Borg , K. Larsson , M. Letzter , and B. M. Sundblad . 2010. “An Index for Breathlessness and Leg Fatigue.” Scandinavian Journal of Medicine & Science in Sports 20(4): 644–650. 10.1111/j.1600-0838.2009.00985.x. 19602182

[ejsc12096-bib-0007] Buchheit, Martin , Hani Al Haddad , Grégoire Paul Millet , Pierre Marie Lepretre , Michael Newton , and Said Ahmaidi . 2009. “Cardiorespiratory and Cardiac Autonomic Responses to 30‐15 Intermittent Fitness Test in Team Sport Players.” The Journal of Strength & Conditioning Research 23(1): 93–100. 10.1519/JSC.0b013e31818b9721. 19057401

[ejsc12096-bib-0008] Castagna, Carlo , Mario Bizzini , Susana C. Araújo Póvoas , Kai Schenk , Gery Büsser , and Stefano D'Ottavio . 2019. “Aerobic Fitness in Top‐Class Soccer Referees.” The Journal of Strength & Conditioning Research 33(11): 3098–3104. 10.1519/JSC.0000000000002264. 29189582

[ejsc12096-bib-0009] Castillo, Daniel , Carlo Castagna , Jesús Cámara , Aitor Iturricastillo , and Javier Yanci . 2018. “Influence of Team’s Rank on Soccer Referees’ External and Internal Match Loads during Official Matches.” The Journal of Strength & Conditioning Research 32(6): 1715–1722. 10.1519/JSC.0000000000002040. 29786628

[ejsc12096-bib-0010] Cohen, Jacob . 1992. “A Power Primer.” Psychological Bulletin 112(1): 155–159. 10.1037/0033-2909.112.1.155. 19565683

[ejsc12096-bib-0011] Dicks, M. , D. O'Hare , C. Button , and D. Rd Mascarenhas . 2009. “Physical Performance and Decision Making in Association Football Referees: A Naturalistic Study.” The Open Sports Sciences Journal 2(9): 1–9. 10.2174/1875399X00902010001.

[ejsc12096-bib-0012] Edwards, S. 1993. “High Performance Training and Racing.” In The Heart Rate Monitor Book, edited by S. Edwards , 113–123. Sacramento CA: Feet Fleet Press.

[ejsc12096-bib-0013] Elsworthy, Nathan , Darren Burke , and J. Ben Dascombe . 2014. “Factors Relating to the Decision‐Making Performance of Australian Football Officials.” International Journal of Performance 14(2): 401–410. 10.1080/24748668.2014.11868730.

[ejsc12096-bib-0014] Emmonds, Stacey , John O'Hara , Kevin Till , Ben Jones , Amy Brightmore , and Carlton Cooke . 2015. “Physiological and Movement Demands of Rugby League Referees: Influence on Penalty Accuracy.” The Journal of Strength & Conditioning Research 29(12): 3367–3374. 10.1519/JSC.0000000000001002. 25970494

[ejsc12096-bib-0015] Eysenck, Michael W. , Nazanin Derakshan , Rita Santos , and Manuel G. Calvo . 2007. “Anxiety and Cognitive Performance: Attentional Control Theory.” Emotion 7(2): 336–353. 10.1037/1528-3542.7.2.336. 17516812

[ejsc12096-bib-0016] Faull, Olivia K. , Pete J. Cox , and Kyle T. S. Pattinson . 2016. “Psychophysical Differences in Ventilator Awareness and Breathlessness between Athletes and Sedentary Individuals.” Frontiers in Physiology 7: 231. 10.3389/fphys.2016.00231. 27378940 PMC4910254

[ejsc12096-bib-0017] Fereday, Kieran , Samuel P. Hills , Mark Russell , Jordan Smith , Dan J. Cunningham , David Shearer , Melitta McNarry , and Liam P. Kilduff . 2020. “A Comparison of Rolling Averages versus Discrete Time Epochs for Assessing the Worst‐Case Scenario Locomotor Demands of Professional Soccer Match‐Play.” Journal of Science and Medicine in Sport 23(8): 764–769. 10.1016/j.jsams.2020.01.002. 31937507

[ejsc12096-bib-0018] Goodall, Stuart , Kevin Thomas , Liam David Harper , Robert Hunter , Paul Parker , Emma Stevenson , Daniel West , Mark Russell , and Glyn Howatson . 2017. “The Assessment of Neuromuscular Fatigue during 120 Min of Simulated Soccer Exercise.” European Journal of Applied Physiology 117(4): 687–697. 10.1007/s00421-017-3561-9. 28247027

[ejsc12096-bib-0019] Hader, Karim , Alberto Mendez‐Villanueva , Said Ahmaidi , Ben K. Williams , and Martin Buchheit . 2014. “Changes of Direction during High‐Intensity Intermittent Runs: Neuromuscular and Metabolic Responses.” BMC Sports Science Medicine Rehabilitation 6(1): 2. 10.1186/2052-1847-6-2. 24417863 PMC3904414

[ejsc12096-bib-0020] International Football Association Board . 2022. Laws of the Game. https://www.theifab.com/document/laws‐of‐the‐game.

[ejsc12096-bib-0021] Kittel, Aden , Nathan Elsworthy , and Michael Spittle . 2019. “Incorporating Perceptual Decision‐Making Training into High‐Intensity Interval Training for Australian Football Umpires.” Journal of Sports Science 37(1): 29–35. 10.1080/02640414.2018.1480257. 29846131

[ejsc12096-bib-0022] Kittel, Aden , Nathan Elsworthy , and Michael Spittle . 2023. “The Effectiveness of above Real Time Training for Developing Decision‐Making Accuracy in Australian Football Umpires.” Research Quarterly for Exercise & Sport 94(1): 64–72. 10.1080/02701367.2021.1939843. 34904910

[ejsc12096-bib-0023] Krustrup, Peter , Werner Helsen , Morten B. Randers , Jesper F. Christensen , Christopher MacDonald , Antonio Natal Rebelo , and Jens Bangsbo . 2009. “Activity Profile and Physical Demands of Football Referees and Assistant Referees in International Games.” Journal of Sports Science 27(11): 1167–1176. 10.1080/02640410903220310. 19705329

[ejsc12096-bib-0024] Lovell, Ric , Sam Halley , Jason Siegler , Tony Wignell , Aaron J. Coutts , and Tim Massard . 2020. “Use of Numerically Blinded Ratings of Perceived Exertion in Soccer: Assessing Concurrent and Construct Validity.” International Journal of Sports Physiology and Performance 15(10): 1430–1436. 10.1123/ijspp.2019-0740. 32987365

[ejsc12096-bib-0025] Mallo, Javier , Pablo Gonzalez Frutos , Daniel Juárez , and Enrique Navarro . 2012. “Effect of Positioning on the Accuracy of Decision‐Making of Association Football Top‐Class Referees and Assistant Referees during Competitive Matches.” Journal of Sports Science 30(13): 1437–1445. 10.1080/02640414.2012.711485. 22867266

[ejsc12096-bib-0026] Mallo, Javier , Enrique Navarro , Jose María Garcia Aranda , and Werner F. Helsen . 2009. “Activity Profile of Top‐Class Association Football Referees in Relation to Fitness‐Test Performance and Match Standard.” Journal of Sports Science 27(1): 9–17. 10.1080/02640410802298227. 18979338

[ejsc12096-bib-0027] Martínez‐Hernández, David , Mark Quinn , and Paul Jones . 2022. “Linear Advancing Actions Followed by Deceleration and Turn Are the Most Common Movements Preceding Goals in Male Professional Soccer.” Science Medicine Football 7(1): 25–33. 10.1080/24733938.2022.2030064. 35062855

[ejsc12096-bib-0028] McEwan, Gary , Rosemary Arthur , Shaun M. Phillips , Neil V. Gibson , and Chris Easton . 2018. “Interval Running with Self‐Selected Recovery: Physiology Performance and Perception.” European Journal of Sport Science 18(8): 1058–1067. 10.1080/17461391.2018.1472811. 29842843

[ejsc12096-bib-0029] McEwan, Gary P. , Viswanath B. Unnithan , Chris Easton , and Rosie Arthur . 2022. “Training Practices and Perceptions of Soccer Officials: Insights from the Referee Training Activity Questionnaire.” International Journal of Sports Science & Coaching 18(4): 1173–1189. 10.1177/17479541221110707.

[ejsc12096-bib-0030] McEwan, Gary P. , Viswanath B. Unnithan , Chris Easton , Andrew J. Glover , and Rosie Arthur . 2023. “Validity and Reliability of the Physiological and Perceptual Responses Elicited during a Novel Treadmill‐Based Soccer Referee Simulation (SRS).” Sport Sciences for Health 19(4): 1153–1161. 10.1007/s11332-023-01043-1.

[ejsc12096-bib-0031] McLaren, Shaun J. , Jonathan M. Taylor , Tom W. Macpherson , Iain R. Spears , and Matthew Weston . 2020. “Systematic Reductions in Differential Ratings of Perceived Exertion across a 2‐week Repeated‐Sprint‐Training Intervention that Improved Players’ High‐Speed‐Running Abilities.” International Journal of Sports Physiology and Performance 15(10): 1414–1421. 10.1123/ijspp.2019-0568. 32678067

[ejsc12096-bib-0032] McMorris, Terry , and Beverley J. Hale . 2012. “Differential Effects of Differing Intensities of Acute Exercise on Speed and Accuracy of Cognition: a Meta‐Analytical Investigation.” Brain and Cognition 80(3): 338–351. 10.1016/j-bandc.2012.09.001. 23064033

[ejsc12096-bib-0033] Nevill, Alan M. , Greg Atkinson , Mike D. Hughes , and S.‐Mark Cooper . 2002. “Statistical Methods for Analysing Discrete and Categorical Data Recorded in Performance Analysis.” Journal of Sports Science 20(10): 829–844. 10.1080/026404102320675666. 12363298

[ejsc12096-bib-0034] Pizzera, Alexandra , Sylvain Laborde , Johannes Lahey , and Patrick Wahl . 2022. “Influence of Physical and Psychological Stress on Decision‐Making Performance of Soccer Referees.” Journal of Sports Science 40(18): 2037–2046. 10.1080/02640414.2022.2127516. 36175198

[ejsc12096-bib-0035] Riiser, Amund , Vidar Andersen , Atle Sæterbakken , Einar Ylvisaker , and Vegard Fusche Moe . 2019. “Running Performance and Position Is Not Related to Decision‐Making Accuracy in Referees.” Sports Medicine International Open 3(2): E66–E71. 10.1055/a-0958-8608. 31428673 PMC6697522

[ejsc12096-bib-0036] Smith, Kurt J. , and Philip N. Ainslie . 2017. “Regulation of Cerebral Blood Flow and Metabolism during Exercise.” Experimental Physiology 102(11): 1356–1371. 10.1113/EP086249. 28786150

[ejsc12096-bib-0037] Sperlich, P. F. , H. C. Holmberg , J. L. Reed , C. Zinner , J. Mester , and B. Sperlich . 2015. “Individual versus Standardized Running Protocols in the Determination of VO2max.” Journal of Sports Science and Medicine 14(2): 386–393: Available at:. https://www.jssm.org/jssm‐14‐386.xml%3EFulltext. 25983589 PMC4424469

[ejsc12096-bib-0038] Spitz, Jochim , Pieter Moors , Johan Wagemans , and Werner F. Helsen . 2018. “The Impact of Video Speed on the Decision‐Making Process of Sports Officials.” Cognitive Research 3(1): 16. 10.1186/s41235-018-0105-8. PMC5994395 29951576

[ejsc12096-bib-0039] Sudo, Mizuki , Joseph T. Costello , Terry McMorris , and Soichi Ando . 2022. “The Effects of Acute High‐Intensity Aerobic Exercise on Cognitive Performance: A Structured Narrative Review.” Frontiers in Behavioral Neuroscience 16. 10.3389/fnbeh.2022.957677. PMC9538359 36212191

[ejsc12096-bib-0040] Weston, Matthew , Carlo Castagna , Franco M. Impellizzeri , Mario Bizzini , A. Mark Williams , and Warren Gregson . 2012. “Science and Medicine Applied to Soccer Refereeing: an Update.” Sports Medicine 42(7): 615–631. 10.2165/11632360-000000000-00000. 22640237

[ejsc12096-bib-0041] Weston, Matthew , Jason Siegler , Andrew Bahnert , James McBrien , and Ric Lovell . 2015. “The Application of Differential Ratings of Perceived Exertion to Australian Football League Matches.” Journal of Science and Medicine in Sport 18(6): 704–708. 10.1016/j.jsams.2014.09.001. 25241705

[ejsc12096-bib-0042] Wiśnik, Piotr , Jan Chmura , Andrzej Wojciech Ziemba , Tomasz Mikulski , and Krystyna Nazar . 2011. “The Effect of Branched Chain Amino Acids on Psychomotor Performance during Treadmill Exercise of Changing Intensity Simulating a Soccer Game.” Applied Physiology Nutrition and Metabolism 36(6): 856–862. 10.1139/h11-110. 22050133

