# Supplementary figures and images for: Decision‐making accuracy of soccer referees in relation to markers of internal and external load
Source: Eur J Sport Sci. 2024 Mar 18;24(6):659–69. doi: 10.1002/ejsc.12096 (PMC11235990; doi:10.1002/ejsc.12096)

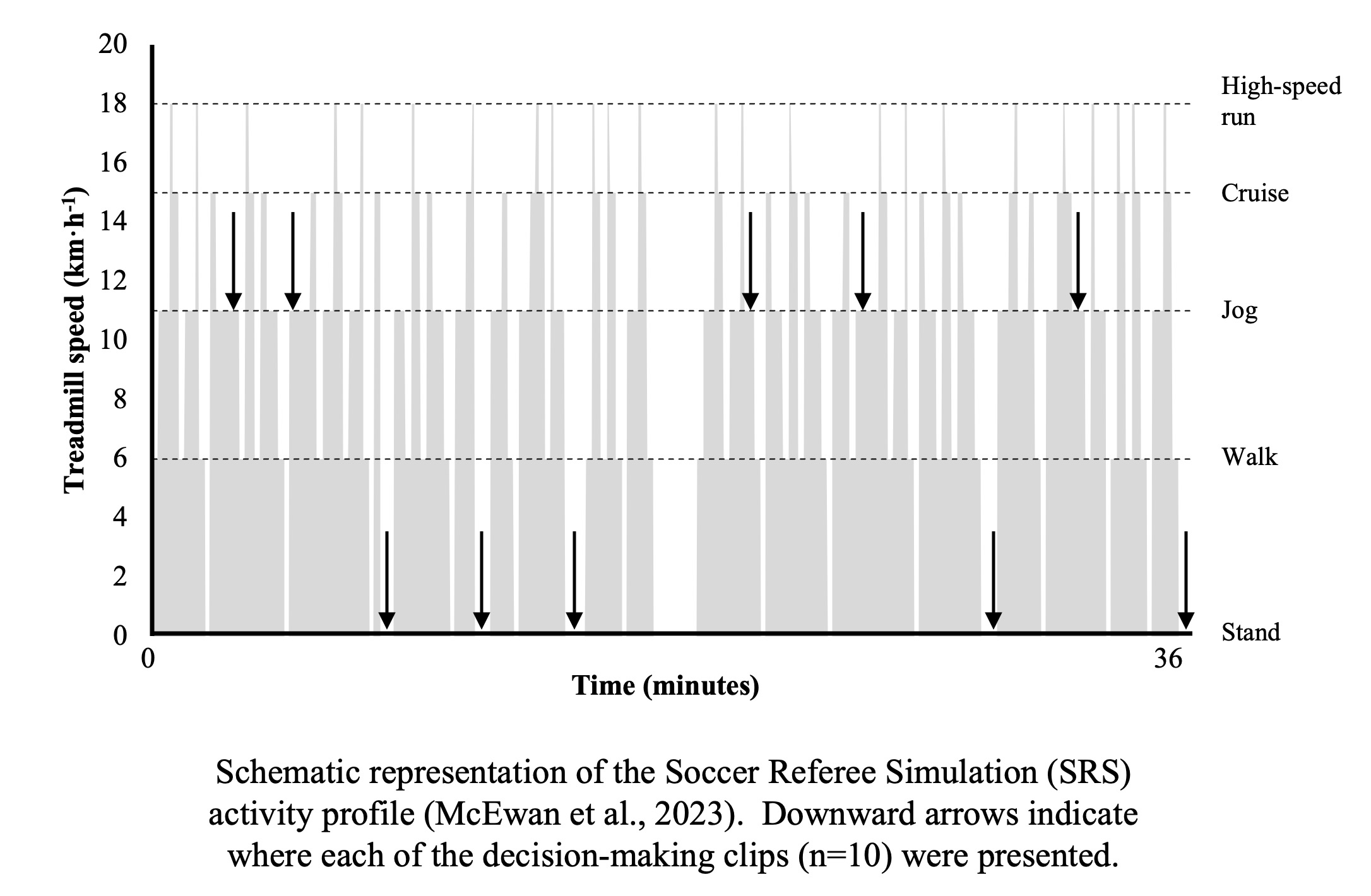

Supplement: Supplementary file 1 — Figure S1 [file EJSC-24-659-s001.jpg]
